# Controlling the oscillation phase through precisely timed closed-loop optogenetic stimulation: a computational study

**DOI:** 10.3389/fncir.2013.00049

**Published:** 2013-04-17

**Authors:** Annette Witt, Agostina Palmigiano, Andreas Neef, Ahmed El Hady, Fred Wolf, Demian Battaglia

**Affiliations:** ^1^Cognitive Neuroscience Department, German Primate Center, Bernstein Center for Computational Neuroscience, Max Planck Institute for Dynamics and Self-OrganizationGöttingen, Germany; ^2^Bernstein Center for Computational Neuroscience, Max Planck Institute for Dynamics and Self-OrganizationGöttingen, Germany; ^3^Max Planck Institute for Dynamics and Self-Organization, Bernstein Center for Computational Neuroscience and Bernstein Focus Neurotechnology, CRC-889 Cellular Basis of Sensory ProcessingGöttingen, Germany

**Keywords:** oscillations, functional connectivity, modeling, closed-loop systems, optogenetic stimulation, phase response

## Abstract

Dynamic oscillatory coherence is believed to play a central role in flexible communication between brain circuits. To test this communication-through-coherence hypothesis, experimental protocols that allow a reliable control of phase-relations between neuronal populations are needed. In this modeling study, we explore the potential of closed-loop optogenetic stimulation for the control of functional interactions mediated by oscillatory coherence. The theory of non-linear oscillators predicts that the efficacy of local stimulation will depend not only on the stimulation intensity but also on its timing relative to the ongoing oscillation in the target area. Induced phase-shifts are expected to be stronger when the stimulation is applied within specific narrow phase intervals. Conversely, stimulations with the same or even stronger intensity are less effective when timed randomly. Stimulation should thus be properly phased with respect to ongoing oscillations (in order to optimally perturb them) and the timing of the stimulation onset must be determined by a real-time phase analysis of simultaneously recorded local field potentials (LFPs). Here, we introduce an electrophysiologically calibrated model of Channelrhodopsin 2 (ChR2)-induced photocurrents, based on fits holding over two decades of light intensity. Through simulations of a neural population which undergoes coherent gamma oscillations—either spontaneously or as an effect of continuous optogenetic driving—we show that precisely-timed photostimulation pulses can be used to shift the phase of oscillation, even at transduction rates smaller than 25%. We consider then a canonic circuit with two inter-connected neural populations oscillating with gamma frequency in a phase-locked manner. We demonstrate that photostimulation pulses applied locally to a single population can induce, if precisely phased, a lasting reorganization of the phase-locking pattern and hence modify functional interactions between the two populations.

## Introduction

Neural activity of brain circuits at many scales has often been reported to display oscillatory components at different frequencies (Eckhorn et al., 1988; Gray et al., 1989; Kreiter and Singer, 1996; Tallon-Baudry et al., 1996; Roelfsema et al., 1997; Varela et al., 2001; Brovelli et al., 2004; Samonds and Bonds, 2004; Melloni et al., 2007; Buffalo et al., 2011). In particular, the *communication-through-coherence* hypothesis (Fries, 2005) suggests that oscillatory coherence between different neural circuits could control functional interactions between them with a high degree of flexibility (Womelsdorf et al., 2007). In particular, evidence for a role of enhanced inter-areal oscillatory coherence in attentional modulation is rapidly accumulating (Fries et al., 2001; Gregoriou et al., 2009; Rotermund et al., 2009; Bosman et al., 2012; Gregoriou et al., 2012; Grothe et al., 2012).

The circuit mechanisms underlying the local generation of oscillations, specifically in the gamma range of frequencies (30–100 Hz) have been explored in studies *in vitro* (Whittington et al., 1995; Bartos et al., 2007) and *in silico* (Brunel and Hakim, 1999; Whittington et al., 2000; Brunel and Hansel, 2006; Wang, 2010). All of these contributions have highlighted the crucial role played by the interplay of GABAergic interneurons in creating time-windows in which excitatory and inhibitory neurons can fire in a sparsely synchronized manner, before being counteracted by strong and delayed feedback inhibition. More recently, the functional involvement of local inhibitory networks could be causally verified *in vivo* by targeted selective optogenetic stimulation of Parvalbumine-positive basket cells in a cortical circuit (Cardin et al., 2006; Sohal et al., 2009).

In an analogous way, optogenetic techniques might be used for direct tests of the communication-through-coherence hypothesis and other suggested functional roles of brain oscillations, like their implication in phase coding (Lisman, 2005; Koepsell et al., 2010; Nadasdy, 2010; Kayser et al., 2012). For such applications, however, improved optogenetic stimulation protocols are needed that allow for precise control of the phase relations between different neuronal populations or assemblies, rather than a pure enhancement of oscillatory power.

Theoretical investigations suggest that, due to non-trivial phase response properties (Pikovsky et al., 2001) of oscillating neuronal populations (Akam et al., 2012), stimulation pulses might have a strong influence on local and long-range phase-relations, but only if properly timed with respect to the ongoing oscillatory dynamics (Tiesinga and Sejnowski, 2010; Battaglia et al., 2012). Application of phase-timed stimuli requires a real-time estimate of the phase from continuously recorded local field potential (LFP) data.

Optogenetic stimulation conditional on recorded activity constitutes a closed-loop setup. The advantage of closed-loop stimulation compared to open-loop stimulation is the possibility to program an artificial feedback with defined rules and constraints dependent on the target system's dynamical history. Closed loop electrical stimulation has been successfully used to clamp network activity (Wallach et al., 2011), to control the firing rate of neurons (Miranda-Dominguez et al., 2010), to control bursting activity (Wagenaar et al., 2005), and to train cultured neuronal networks (Marom and Shahaf, 2002). Closing the loop between living neurons and robotics has also been used to realize embodiment—by using representations generated by network activity either to control a robotic arm (Bakkum et al., 2007) or control autonomous systems (Bandyopadhyay, 2005)—or to study neuronal plasticity (Novellino et al., 2007).

In this study, we explore through a modeling approach the feasibility of closed-loop optogenetic control of the phase of a local oscillation and of inter-areal phase synchronization. To this end, we simulated the activity of populations of excitatory and inhibitory conductance-based neurons with random connectivity. To investigate the case where a sparse transduction with Channelrhodopsin 2 (ChR2) is achieved *in vitro* or *in vivo*, small fractions of these neurons were endowed with a newly developed and data-constrained conductance-based model of a light-activated channel. This case is of particular interest, since it has been shown that low transduction rates achieved through either particle mediated gene transfer or via lipid reagents (Takahashi et al., 2012) can increase the spatial specificity of light stimulation (Schoenenberger et al., 2008). Our model, however, applies robustly also to the case of higher ChR2 transduction rates, as the ones that can be achieved using viral transfection (Adamantidis et al., 2007; Aravanis et al., 2007), in utero electroporation (Petreanu et al., 2007) or in T helper type 1 (Thy1) transgenic mice (Wang et al., 2007).

Demonstrating the reliability of our model, we first simulated phase shifting of LFP oscillations with open-loop optogenetic stimulation, quantitatively reproducing and generalizing experimental results *in vitro* (Akam et al., 2012). We moved then to the analysis of a canonical cortical circuit with two interacting areas. Here, we simulated a realistic closed-loop stimulation protocol which was suited to trigger lasting changes of inter-areal phase relations and, correspondingly, to affect communication-through-coherence. Thus, we intend our modeling exploration to foster the implementation of a new generation of closed-loop optogenetic experiments *in vitro* and *in vivo* aiming at inducing distributed reorganization of functional interactions at the system level.

## Materials and methods

### ChR2 photocurrent experimental characterization

**Figure 1:**
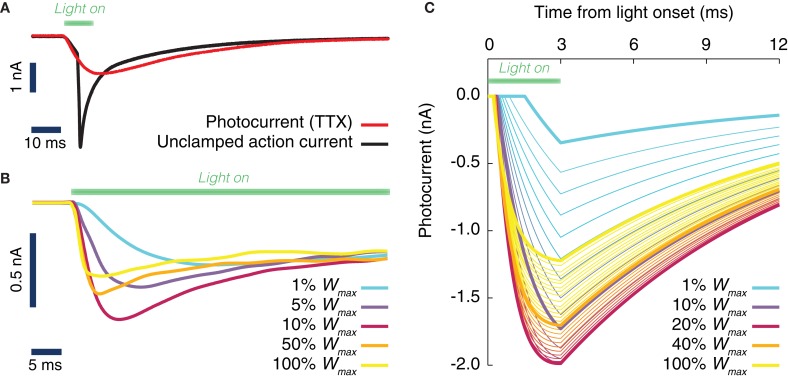
**Evoked ChR2 photocurrent: conductance-based model. (A)** Whole cell voltage clamp recording of a cultured neuron, transduced with Channelrhodopsine 2 (ChR2) and illuminated with LED light (during the time interval shown by a green horizontal bar). Two current intensity recordings have been performed, the first in a physiological solution, i.e., with all channels active (black curve), the second with TTX in the bath, i.e., with blocked Na-channels (red curve). When the Na channels are still active (black curve), even the voltage clamp (−70 mV) at the soma cannot prevent the cell from spiking. **(B)** Activation kinetics of the photo-induced conductance in human embryonic kidney cells (HEK-293) that are transfected with ChR2. For increasing light power density (100% *W*_max_ corresponds approximately to 130 mW/mm^2^) the activation becomes faster. Peak conductance increases from 0 to ~10% of the maximal intensity and decreases for higher light intensities. Note the different scale of evoked currents in neurons and HEK cells. **(C)** Simulated photocurrents generated by the conductance-based model described by Equation (1), for different light intensities (expressed relatively to maximum illumination intensity) and for a rectangular shaped light pulse stimulation with a duration of 3 ms. Model parameters and their dependence on light intensity (see Table 2) are obtained from fits to photoconductance recordings analogous to the one shown in panel **(B)**, performed for different light intensities. For short light pulses as used here, the experiments indicate that the largest conductances are obtained for light intensities between 10% and 50% (interpolation of the simulated photocurrent results in an optimal value of 18% of the maximum light intensity).

Human embryonic kidney cells were transfected with a plasmid encoding a ChR2-YFP fusion protein. The pcDNA 3.1-ChR2-YFP construct was kindly provided by Ernst Bamberg, (MPI for Biophysics, Frankfurt, Germany). After two–four days, successfully transfected cells were identified by their YFP fluorescence. In the whole-cell configuration, the membrane voltage was clamped to −60 mV. Channelrhodopsin's conductance was changed by 500 ms long light pulses. The conductance change was monitored as a time and light-intensity dependent current change (Figure 1B). In the case of cultured hippocampal neurons, cell were transfected at 7 DIV with AAV1/2-CAG-ChR2-YFP virus. After 1 week, successfully transduced cells could be identified by their YFP fluorescence.

Whole-field illumination was provided by an extended laser beam (488 nm). Light intensity was controlled by neutral density filters (optical density 1 and 2, respectively) and by means of the software provided for the laser. A comparison of the light-induced current waveforms for 90% attenuation by software and a neutral density filter with an optical density of 1.0 showed excellent agreement, indicating that the software produced the intended attenuation. The laser was switched using a built-in mechanical shutter with a response time in the μs range, achieved through the minute spatial extent of the beam.

### Biophysically calibrated model of ChR2 photoconductance

Based on the results of the previously described experiment, we modeled the evoked photocurrents as the product of activation and inactivation functions. The current activation could be described by a single exponential function and the current inactivation by the sum of two exponential functions (see also Figure 1B). This light-induced conductance change could be well described by the functional form:

(1) $$F_{ChR2}(t)=A_{\text{act}}\left(1-e^{-\frac{t-t_{\text{ON}}-d}{\tau_{\text{act}}}}\right)\;\cdot \;\left(A_{\text{persist}}+A_{\text{inact}}^{\left(1\right)}e^{-\frac{t-t_{\text{ON}}-d}{\tau_{\text{inact}}^{\left(\text{1}\right)}}}+A_{\text{inact}}^{\left(2\right)}e^{-\frac{t-t_{\text{ON}}-d}{\tau_{\text{inact}}^{\left(2\right)}}}\right)$$

Here *d* represents a latency observed between the times *t*_ON_ of light onset and the actual start of the conductance rise and *A*_persist_ is set to *A*_persist_ = 1 − *A*^(1)^_inact_ − *A*^(2)^_inact_ in order to prevent the inactivation conductance factor from becoming negative. Note that Equation 1 holds true only as long as the light is switched on. After switching off the light, the response returns to baseline with a single exponential time course with time constant τ_OFF_. When individual current responses were fitted, the latency *d*, the amplitude *A*_act_, the inactivating fractions *A*^(1)^_inact_ and *A*^(2)^_inact_, and the activation time constant τ_act_ were found to be dependent on the light-intensity *W*_light_ when individual current responses were fitted. However, the time constants related to inactivation were almost unchanged for different light intensities. Therefore, for simultaneously fitting current responses evoked by different light intensities (ranging over two orders of magnitude), two global (i.e., light-independent) parameters τ^(1)^_inact_ and τ^(2)^_inact_ were used. In order to model the dependence on the light intensity of the other parameters (*d*, τ_act_, *A*_act_, *A*^(1)^_inact_, and *A*^(2)^_inact_) we fitted the following functions to the recorded data:

(2) $$d=d_{A}+d_{B}W_{\text{light}}+\frac{d_{C}}{W_{\text{light}}}$$

(3) $$\tau_{\text{act}}=\tau_{\text{act}}^{\left(0\right)}+c_{\text{act}}e^{-k_{\text{act}}W_{\text{light}}}$$

(4) $$A_{\text{act}}=a_{0}+\frac{a_{\text{min}}-1}{1+{\left(W_{0.5}/W_{\text{light}}\right)}^{2}}$$

(5) $$A_{\text{inact}}^{\left(1\right)}=b_{0}+\frac{b_{1}}{b_{2}+{\left(W_{\text{light}}-W_{\text{inact}}\right)}^{2}}$$

(6) $$A_{\text{inact}}^{\left(2\right)}=c_{\text{inact}}e^{-k_{\text{inact}}W_{\text{light}}}$$

All the parameters of Equations (2–5) are the result of least-squared fits. For Equation (6) *k*_inact_ has been set manually to assure convergence of the fitting procedure. All fitted parameters of the ChR2 conductance model, together with their standard deviations, are summarized in Table 1. Light intensity is measured relatively to the maximum intensity *W*_max_ that can be achieved in our setup. A precise calibration of the absolute power density at the maximal intensity was not performed. We have estimated it to be approx. *W*_max_ = 130 mW/mm^2^ for a continuous illumination, which is rather high if compared to 5–6 mW/mm^2^ used by Ishizuka et al. (2006) and Ernst et al. (2008) and the maximum (around 20 mW/mm^2^) used in Nikolic et al. (2009).

**Table 1:** **Parameters of the ChR2 conductance model**.

**Type of parameter**	**Parameter**	**Value ± *SD* l.sq fit (unit)**
Latency	*d*_*A*_	0.27 ± 0.04 (*ms*)
	*d*_*B*_	−0.05 ± 0.06 (*ms*/[*W*_light_])
	*d*_*C*_	0.0126 ± 0.0006 (*ms* × [*W*_light_])
Activation	τ^(0)^_act_	0.74 ± 0.20 (*ms*)
	*c*_act_	12.0 ± 0.4 (*ms*)
	*k*_act_	25 ± 2 (1/[*W*_light_])
	*a*_0_	1.00 ± 0.04
	*a*_min_	0.4 ± 0.1
	*W*_0.5_	0.38 ± 0.15 ([*W*_light_])
Inactivation (first component)	τ^(1)^_inact_	9.06 (*ms*)
	*b*_0_	0.16 ± 0.01
	*b*_1_	0.013 ± 0.004 ([*W*_light_]^2^)
	*b*_2_	0.027 ± 0.007 ([*W*_light_]^2^)
	*W*_inact_	0.11 ± 0.01 ([*W*_light_])
Inactivation (second component)	τ^(2)^_inact_	59.6 (*ms*)
	*c*_inact_	0.29
	*k*_inact_	2.4 (1/[*W*_light_])
Deactivation	τ_off_	10 (ms)
Coupling prefactor	*g*_*ChR2*_	0.007 (μ S)

*Parameters to simulate time and light-intensity dependent conductance changes mediated by channelrhodopsin 2. Errors are sample standard deviations. Parameters returned from the global fit procedure do not have a measure of uncertainty. See section Materials and methods for the model description*.

### ChR2-transduced neuronal populations model

A local neuronal population was modeled as a random network of *N*_*E*_ = 4000 excitatory and *N*_*I*_ = *N*_*E*_/4 = 1000 inhibitory conductance-based model neurons of the Wang-Buzsáki (WB) type (Wang and Buzsáki, 1996). The WB model describes a single compartment neuron endowed with sodium and potassium currents. The membrane potential follows the equation:

(7) $$C\frac{dV}{dt}=-I_{L}-I_{Na}-I_{K}+I_{\text{syn}}+I_{\text{noise}}+\kappa I_{ChR2}$$

where *C* is the capacitance of the neuron, *I*_*L*_ = *g*_*L*_ (*V* − *V*_*L*_) is the leakage current, *I*_syn_ reflects recurrent interactions with other neurons in the network, *I*_noise_ models the driving exerted by background noise and *I*_*ChR2*_ is the photocurrent-induced by external light stimulation. Sodium and potassium currents are voltage-dependent and given by *I*_*Na*_ = *g*_*Na*_*m*^3^_∞_*h*(*V* − *V*_*Na*_) and *I*_*K*_ = *g*_*K*_*n*^4^(*V* − *V*_*K*_). The activation of the sodium current was modeled as instantaneous. We used sodium and potassium current voltage-dependent activation and inactivation functions as given in Wang and Buzsáki (1996).

The synaptic current evoked by a single presynaptic action potential was given by *I*_spike_(*t*) = −*g*_α_*s*_spike_(*t*)(*V* − *V*_α_), where the reversal potential *V*_α_ of the synapse is 0 mV for excitatory AMPA synapses (α = *E*) and −80 mV for inhibitory GABA synapses (α = *I*). The time-course of the postsynaptic conductance was described as a difference of exponentials:

(8) $$s_{\text{spike}}(t)\propto \left(e^{-\left(t+d_{\text{syn}}-t_{\text{spike}}\right)/\tau_{\text{rise}}}-e^{-\left(t+d_{\text{syn}}-t_{\text{spike}}\right)\tau_{\text{decay}}}\right)$$

for *t* > *t*_spike_, 0 otherwise, where *t*_spike_ is the time of the presynaptic spike, *d*_syn_ is a combined conduction and synaptic delay, and τ_rise_ and τ_decay_ are respectively the rise- and decay time constants. The normalization constant of *s*_spike_(*t*) was chosen such that its peak value is equal to 1. The peak conductances of all excitatory and inhibitory synapses were set to *g*_*E*_ and *g*_*I*_, respectively. The total recurrent current *I*_syn_(*t*) was then given by the sum of the contributions *I*_spike_(*t*) from all presynaptic spikes fired before time *t*.

The background noise input *I*_noise_ to each neuron was modeled as an additional synaptic current-induced by statistically independent Poisson trains of excitatory spikes with a common firing rate ν_noise_ and a peak conductance *g*_noise_.

Excitatory and inhibitory neurons in the populations were transduced by ChR2 with a same probability, given by the transduction rate *P*_*ChR2*_. The photocurrent prefactor κ was set to 1 and 0 respectively for transduced and non-transduced neurons. The induced photocurrent was given by *I*_*ChR2*_(*t*) = −*g*_*ChR2*_*F*_*ChR2*_ [*W*_light_(*t*)](*V* − *V*_*ChR2*_). The conductance waveform *F*_*ChR2*_(*t*) given by Equation (1)—that depends on the applied waveform *W*_light_(*t*) of the optical stimulation—was multiplied by a prefactor *g*_*ChR2*_, such that the peak photocurrent evoked by a pulse with optimal light intensity in the used model neurons (simulated at resting potential) was 2 nA. The reversal potential was *V*_*ChR2*_ ≅ 0.

Excitatory neurons established synapses with other excitatory or inhibitory neurons within the same local circuit with probability *P*_*E*_, inhibitory neurons with probability *P*_*I*_. In addition, when considering multiple interconnected local areas, excitatory neurons within a local circuit established long-range connections with excitatory or inhibitory neurons in a remote local area with a probability *P*^(*lr*)^_*E*_.

### Adopted parameters and oscillatory synchrony

The neuronal population model described in the previous section can generate two qualitatively different dynamical regimes, characterized by different degrees of oscillatory coherence. The network resides in one or the other regime depending both on the drive to the network, controlled in this study by varying the background firing rate ν_noise_, and on the strength of local inhibitory interactions, controlled in this study by varying the probability of inhibitory connection *P*_*I*_.

The single neuron and network parameters used for all simulations are summarized in Table 2. However, we note that qualitatively similar dynamical features, in particular the existence of a smooth transition between a weakly and a strongly synchronous oscillatory regime, would be obtained for a broad range of parameters, with the frequency of the collective oscillation primarily determined by the synaptic time constants, τ_rise_ and τ_decay_, (Brunel and Wang, 2003). We also find that the transition toward strong synchrony tends to get sharper with increasing network size [not shown, but see as well (Brunel and Hakim, 1999)].

**Table 2:** **Parameters of the spiking neuronal network model**.

**Type of parameter**	**Parameter**	**Value (unit)**
Single neuron	*g*_*L*_	0.01 (μS)
	*V*_*L*_	−65 (mV)
	*C*	100 (pF)
	*g*_*Na*_	3.5 (μS)
	*V*_*Na*_	55 (mV)
	*g*_*k*_	0.9 (μS)
	*V*_*k*_	−90 (mV)
	*m*_∞_, *h*, *n*	See Wang and Buzsáki (1996)
Population size	*N*_*E*_	4000
	*N*_*I*_	1000
Excitatory synapses	τ_rise_	1 (ms)
	τ_decay_	3 (ms)
	*g*_*E*_	0.5 (μS)
Inhibitory synapses	τ_rise_	1 (ms)
	τ_decay_	4 (ms)
	*g*_*I*_	18 (μS)
Synaptic latencies	*d*_syn_ (local)	1.5 (ms)
	*d*_syn_ (long-range)	1.0 (ms)
Connection probabilities	*P*_*I*_	0.3
	*P*_*E*_	0.12
	*P*^(*lr*)^_*E*_	0.06
Background noise	*v*_noise_	3 (kHz)
	*g*_noise_	0.5 (μ S)

*Parameters to simulate the activity of transduced neuronal populations (see section Materials and methods for the model description)*.

Synchronization of the population activity was quantified through the synchronization index χ (Golomb and Hansel, 2000):

(9) $$\chi =\frac{\sigma_{\text{LFP}}^{2}}{\langle \sigma_{V_{i}}^{2}\rangle }$$

given by the ratio between the variance of the average membrane potential of all excitatory and inhibitory neurons in the local population—here briefly defined conventionally as the “LFP” signal—and the average variance of the membrane potentials *V*_*i*_ of individual neurons in the population. The synchronization index χ is bounded in the unit range, χ = 0 meaning asynchronous and χ = 1 fully synchronous dynamics.

The dependency of firing rate of excitatory and inhibitory neurons, of the collective oscillation frequency and of the synchrony level χ was studied by systematically varying the parameters ν_noise_ in the range between 2 and 6 kHz and *P*_*I*_ between 0.2 and 0.6 (the reference values, tabulated in Table 2, being ν_noise_ = 3 kHz and *P*_*I*_ = 0.3). All the quantities were evaluated over simulated time-series lasting 20 s of real time.

### Analysis of phase response

Although the simulation generates spike trains for all neurons, we focus here on alterations of the ongoing collective activity and, therefore, on the oscillating LFP signal. A single rectangular-shaped light pulse with a given intensity *W*_light_ and duration *T*_light_ was applied to the considered network at a specific time of application *t*_ON_. For different values of *W*_light_ and *T*_light_, we tested the effects of overall 1500 different light onset times *t*_ON_, distributed uniformly over a time interval of approximately 50 oscillation periods. Indeed, averaging over multiple periods was required, because of stochastic fluctuations of the period length. For each stimulation pulse, the activity of the network was further simulated over 60 oscillation cycles following the perturbation.

In every simulation run, the initial conditions, the network topology and the background noise were kept identical, in order to exclusively study the dependence of the induced perturbation on the parameters of the light stimulation and its application time. Pairs of LFP time series were thus generated consisting of a time series after the application of a photostimulation and a time series of the corresponding unperturbed neural dynamics. For every such pair of time series, instantaneous phase values were extracted using a Hilbert transform (Gabor, 1946), an approach extensively used for investigating phase dynamics and synchronization of non-linear oscillators (Pikovsky et al., 2001). The induced phase shift was then measured by averaging the phase difference Δϕ between the perturbed and the unperturbed LFPs over the last 50 recorded oscillation cycles. A transient of 10 oscillation cycles immediately following *t*_ON_ was discarded to ignore transient effects caused by the applied light pulse. The times of perturbation application *t*_ON_ were translated into phases and binned into 30 equally sized phase bins. The observed phase shifts Δϕ were averaged over each bin and plotted as a function of the phase of perturbation application ϕ(*t*_ON_) for different light intensities *W*_light_ and perturbation pulse duration *T*_light_, and also for networks with different transfection rates *P*_*ChR2*_.

The dependency of phase responses on varying values of light intensity, pulse duration and timing of the perturbation were investigated for a specific realization of the network random connectivity. We have repeated our analysis for three different random realizations of connectivity (with the same homogeneous probabilities of connection, *P*_*I*_ and *P*_*E*_). The corresponding phase responses to light stimuli were qualitatively and quantitatively very similar (not shown). In particular, differences between random network instances were of the same order of magnitude as the error bars shown in Figure 4, corresponding to fluctuations of the phase response over time for a same connectivity realization. These similarities are not surprising and match theoretical expectations, since dynamical effects arising from fluctuations due to finite-size connectivity are small for the large network size adopted here (Golomb and Hansel, 2000). Therefore, we can conclude that our results hold in general for random networks with the same (in a probabilistic sense) connectivity features.

### Analysis of phase locking changes

If two coupled neuronal populations are simulated with the parameters given in Table 2, the oscillations of the two LFPs self-organize in a phase-locked configuration. The temporarily stable relative phase difference, Δϕ, can have two different values: Δϕ_locked_ or 1 − Δϕ_locked_ (phases are measured over the cyclic unit interval 0 ≤ ϕ ≤ 1). Both phase-locking values correspond to out-of-phase configurations in which either of the two populations leads in phase over the other.

In our simulations, only one of the two local neuronal populations was transduced with ChR2. We applied light stimulation pulses to this transduced population, with a light intensity *W*_light_ = 20% (expressed as the percentage of the maximum possible light intensity of our setup *W*_max_) and a pulse duration of *T*_light_ = 3 ms. Similar to the protocol used for the phase response analysis of a single population, 1500 different pulse onset times, *t*_ON_, were used, which were uniformly distributed over 50 oscillation cycles. Starting from random initial conditions, no perturbation was applied for the first 100 oscillation cycles, to ensure complete convergence to a stable phase-locked attractor. Without loss of generality, we considered the configuration in which the phase of the transduced population leads over that of the not transduced population (i.e., in which the stable inter-circuit phase difference is close to Δϕ_locked_ before the perturbation).

Variations of the phase-difference between the two populations were measured in two different time-windows. We first studied the short-term effects of the light stimulation, by averaging the instantaneous Hilbert phase difference over the first 5 oscillation cycles after the perturbation. Binning different onset times according to the corresponding phase of application of the perturbation (as done for the estimation of single population phase response), we quantified the probability *P*_shifting_(ϕ), that a light pulse induces a relative variation of more than 10% (reduction or increase) of the inter-population phase-difference. For each application phase bin, *P*_shifting_(ϕ) was compared with the probability of observing similarly large spontaneous fluctuations of Δϕ in the unperturbed activity of the same network.

We then studied longer term effects of the light stimulation by averaging the difference of the instantaneous Hilbert phases over the 50 cycles that follow the ten omitted oscillation cycles directly after stimulation. The aim of this long-term analysis was to assess the occurrence of a switching from the phase-locking pattern with phase-difference close to Δϕ_locked_ toward the other phase-locking pattern with phase difference close to 1-Δϕ_locked_. Once again binning onset times according to the corresponding phase of perturbation application, we quantified the probability *P*_switching_(ϕ) that the long-term averaged phase difference was within a tolerance interval of 1 − Δϕ_locked_ ± δ, with δ = 0.05 (i.e., the transduced population switched steadily from the role of phase leader to phase laggard). For each phase bin, *P*_switching_(ϕ) was compared to the probability of observing a spontaneous switching of the phase locking (from Δϕ_locked_ to 1-Δϕ_locked_) over an equivalent time span of 50 cycles, based on time-series of the unperturbed dynamics of the same network.

The probabilities *P*_shifting_(ϕ) and *P*_switching_(ϕ) were finally plotted as polar histograms with ten equally-spaced bins for the phase of the onset of the light stimulation ϕ(*t*_ON_), in which the corresponding probabilities of spontaneous shifting or switching were also reported in order to identify phase bins in which the effects induced by the perturbation pulse were significantly low or high (Figure 5).

### Online phase prediction

A closed-loop approach (Figure 6) is necessary to estimate a time *t*_ON_ which corresponds to a future occurrence of a given target phase ϕ_target_, leading to the largest possible probability of switching of the inter-areal locking (Figure 5).

To study the feasibility of such an approach, we modeled its implementation, considering the same bi-areal network used to characterize induced switching between phase-locked states (see previous section and Figure 5). Simulated LFPs were recorded from both the stimulation target area and a second coupled area. However, the calculations performed online involved only the LFP time-series *V*(*t*) recorded in the target area. The time-series $\tilde{V}(t)$ of the second area were recorded and analyzed offline to determine phase-locking patterns before and after the stimulation.

We approximated the “true” Hilbert phase ϕ_*H*_(*t*) associated to *V*(*t*) by a linearly interpolated phase. This approximation could be simply done by interpolating a variable ϕ_*L*_(*t*)that was linearly growing in the unit interval 0 ≤ ϕ_*L*_ < 1 between any two times *t*_*k*_ and *t*_*k* + 1_ delimiting an oscillation cycle. As shown by Figure 7B, the phase variables ϕ_*H*_(*t*) and ϕ_*L*_(*t*) are related by a mildly non-linear map, described as a static non-linearity ϕ_*H*_ = *f*_LH_ (ϕ_*L*_). However, we systematically ignored this non-linearity in the following by approximating ϕ_*H*_(*t*) directly by ϕ_*L*_(*t*).

The workflow for the prediction of the perturbation onset time *t*_ON_ is split up into multiple stages (Figure 6). First of all, it was necessary, during a *testing stage*, to detect the presence of sufficiently strong local oscillations and to measure their average frequency *f*_peak_. It was important to monitor the characteristics of LFP oscillations (band-passed around *f*_peak_) in the stimulation target area (*monitoring stage*) and to extract, based on observations of past activity, a model able to approximately predict future phase evolution (*prediction stage*).

Even in the ideal case of an elevated synchrony index χ and sustained oscillations, the duration of oscillation periods *T*_*i*_ fluctuated from cycle-to-cycle around their average $\bar{T}$(cf. Figure 7A). Let us suppose that the last oscillation period recorded in the monitoring stage was *T*_*k*_ = *t*_*k*_ − *t*_*k* − 1_ and that the prediction stage lasts (less than) *s* oscillation cycles. Neglecting correlations between period lengths of consecutive cycles, the time of beginning of the next cycle after the end of the prediction stage could be estimated via a simple *linear extrapolation*:

(10) $$t_{k+s}^{\left(0\right)}=t_{k}+s\bar{T}$$

However, for our network model, the temporal autocorrelation function of period lengths *T*_*i*_, *i* = 1, …, *k* displayed a fast but not instantaneous decay for increasing lags (measured in oscillation cycles). These weak, positive correlations between consecutive cycle durations could be well captured by a *first order autoregressive process* [AR(1)], $T_{i}=\bar{T}+a(T_{i-1}-\bar{T})+\epsilon_{i}$, with $\bar{T}$ the average oscillation period over the monitoring time-window, *a* the AR(1) coefficient and _*i*_ an i.i.d. Gaussian distributed residual noise term (Brockwell and Davis, 1996). With this AR(1) model, the beginning of the next cycle was estimated as:

(11) $$t_{k+s}^{\left(1\right)}=t_{k}+s\bar{T}+\left(\frac{a^{s+1}-a}{a-1}\right)\;\cdot \;T_{k}$$

The AR(1) coefficient was derived as:

(12) $$a=\frac{k}{k-1}\frac{\sum_{i=1}^{k-1}\left(T_{i}-\bar{T}\right)\left(T_{i+1}-\bar{T}\right)}{\sum_{i=1}^{k}{\left(T_{i}-\bar{T}\right)}^{2}}$$

based on the periods *T*_*i*_, *i* = 1, …, *k*, measured during the monitoring stage and on their average duration $\bar{T}$.

Spectral analysis of LFPs recorded in the stimulation target area and in a second coupled area was performed during the testing stage. A windowed Fast Fourier Transform (FFT) was applied to demeaned chunks of the LFP signal, to extract a rough estimate of the instantaneous power spectrum. When the power at some frequency *f*_peak_ in the gamma range exceeded a determined threshold in both recorded areas, the monitoring stage started.

During the monitoring stage, a computationally efficient low-order recursive time domain filter (Percival and Walden, 1993) was applied to clean the oscillating LFP signals. The filtered time-series was computed online as:

(13) $$V_{\text{filtered}}(t)=V(t)+\alpha_{1}V_{\text{filtered}}(t-1)+\alpha_{2}V_{\text{filtered}}(t-2)$$

Filter coefficients were chosen as α_2_ = −0.99 and α_1_ = 4α_2_ cos(2π(1 − *f*_peak_))/(1 − α_2_) (assuming a sampling rate of 1 kHz). The pass frequency was then equal to *f*_peak_ and the main frequency of the activity of recorded areas was maintained. The LFP time-series *V*(*t*) and $\tilde{V}(t)$ recorded during the monitoring stage were stored. An analysis of the inter-areal phase-locking pattern before stimulation was then performed offline, while the closed-loop experiment was continuing. A monitoring stage including approximately 20 oscillation cycles was found to be sufficiently long to achieve accurate model estimation.

The limited amount of fast computations to be performed during the prediction stage is summarized as follows:

1. Subtract the mean value from the band-passed LFP time series *V*_filtered_(*t*) measured during the monitoring window in the stimulation target area.
2. Calculate the timings *t*_0_, *t*_1_, …, *t*_*k*_ at which the LFP *V*_filtered_(*t*) crosses zero. Their differences *T*_*i*_ = *t*_*i*_ − *t*_*i* − 1_, *i* = 1, …, *k* are the estimated period lengths of the observed oscillations.
3. Calculate the average period length $\bar{T}$ from the series of *T*_*i*_ .
4. If the AR(1) approach is used, then compute the *a* coefficient based on equation (12) and compute the perturbation onset time as $t_{\text{ON}}^{\left(1\right)}=t_{k+s}^{\left(1\right)}+\phi_{\text{target}}\bar{T}$, where *t*^(1)^_*k* + *s*_ is given by Equation (11).
5. If a simpler linear extrapolation is used, compute the perturbation onset time directly as $t_{\text{ON}}^{\left(0\right)}=t_{k+s}^{\left(0\right)}+\phi_{\text{target}}\bar{T}$, where *t*^(0)^_*k*+ *s*_ is given by Equation (10).

After the application of the perturbation pulse, the LFPs of both areas were recorded and stored. An analysis of the inter-areal phase-locking pattern after stimulation was then performed offline and compared to the phase-locking assessed before stimulation. In case of failed switching, either the same linear model was used to extrapolate directly the time *t*_ON_ of a further stimulation pulse, or a new testing stage was initiated, verifying that oscillations were still ongoing or waiting for the next oscillatory epoch to begin.

The decision between a prediction scheme based on the AR(1) model and a simpler linear extrapolation scheme depends ultimately on the correlation statistics of the series of period lengths. It can be shown that the prediction error of the estimated phase is reduced by the AR(1) prediction scheme compared to linear extrapolation by a maximal amount of $100\%/\sqrt{1-a^{2}}$ (and by exactly this amount for Gaussian distributed samples). If the AR(1) parameter *a* estimated from the recordings during the monitoring window is small (as a rule of thumb, *a*<0.3), then the performance improvement is negligible and advantage can be taken from the faster computation of the simpler linear extrapolation. Unfortunately, this criterion requires the evaluation of *a*. Nevertheless, the analysis of Figure 7E indirectly suggests that the AR(1) coefficient depends non-monotonically on the synchrony level, and that it increases going from low to intermediate synchrony indices χ, but drops again going toward higher χ. The choice of a high power threshold during the testing stage guarantees a high level of synchrony and, therefore, small values of *a* during the monitoring stage. This allows one to adopt the computationally faster step (5) instead of (4). However, a tradeoff should be made between the need of a fast prediction and the probability to detect a number of oscillatory epochs sufficient for meeting the testing stage criteria.

## Results

### Data-constrained model of ChR2-photocurrent

In order to assess from *in silico* experiments the efficacy of optogenetic stimulation in inducing changes of local phase or of inter-areal phase relations, we first derived a realistic and fully data-constrained model of the evoked ChR2 conductance. To do so, we first performed an experimental characterization of photocurrents evoked in living cells *in vitro* by light stimulation over a broad range of light intensities spanning two decades of power (see section Materials and Methods). Then, based on this systematic set of measurements, we fitted to the whole dataset a unique conductance-based model that describes the evoked time-dependent photocurrent, and hence the conductance, as the product of activation and inactivation factors.

The light-activated ChR2 ion channel mediates a current that is carried mostly by Na^+^, K^+^, and H^+^ with contributions of Ca^2+^. Its reversal potential is typically around 0 mV and therefore it is depolarizing at neuronal resting potential. We found that upon illumination onset, a current built up with a nearly exponential time course with a time constant τ_act_ ranging from 10 ms, for very weak light intensities that barely evoked any current response, to below 1 ms for high intensities. For a large range of intensities the current displayed a transient behavior and its amplitude, after reaching a peak, decayed over tens of milliseconds to reach a plateau. This inactivation behavior was biphasic and its time constants were not dependent on light intensity, unlike the activation time constant. Finally, when the light was switched off, the current decayed back to baseline with a time course that was well described by a single exponential with a 10 ms time constant.

Figure 1A depicts inward currents induced by a light pulse of moderate intensity (approximately 3 mW/mm^2^ for 10 ms) in a cultured hippocampal neuron transduced with ChR2. Even such a weak light pulse was able to elicit an action current, as the axon escaped the voltage-clamp (Figure 1A, black line). The ChR2 photocurrent could be isolated, by blocking Na-channels with tetrodotoxin (Figure 1A, red line).

To achieve an improved characterization of the photocurrent time-course, we systematically analyzed recordings over (non-spiking) transfected kidney cells (Figure 1B) using a very large range of light power densities for the characterization of ChR2 activation and inactivation kinetics. We found that the peak and steady state photocurrent do not increase monotonically with light power density. A maximal peak current is achieved around 10–20% of the maximum power density (see section Discussion). For applications, where such power densities can be attained, for instance with a laser or a strongly focused LED, a careful tuning of the applied light intensity could thus potentially reduce the minimum transduction rate needed to efficiently drive the local oscillations in a target area.

As detailed in section Materials and Methods, it was possible to capture the time-course of the evoked ChR2 current with a single conductance-based model with light-dependent parameters. The simulated photocurrents generated by the model in response to a single square pulse of light lasting 3 ms are shown in Figure 1C for various light intensities (corresponding to the typical short pulse length used in the simulations of next sections). As evident from Figure 1C, our data-constrained model was able to capture the non-monotonic dependence of peak photocurrent on the light intensity, leading to the largest peak photocurrent for a light intensity of approximately 18% the largest deliverable intensity *W*_max_ .

### Spiking network models of transduced oscillating areas

**Figure 2:**
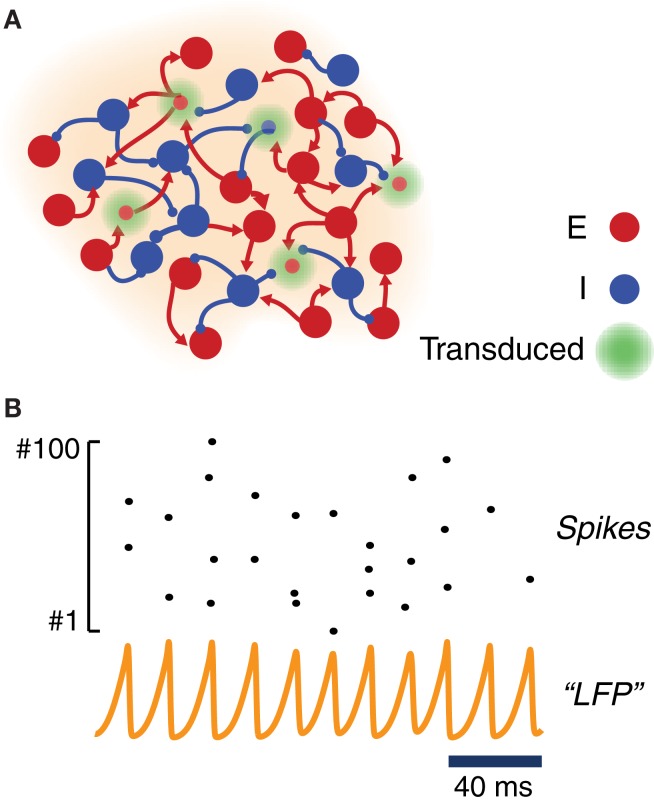
**Model of a ChR2-transduced population. (A)** Graphic cartoon of a randomly-connected network of inhibitory and excitatory spiking neurons. In order to model the effects of local ChR2 transduction a variable fraction of the neurons is endowed with a ChR2 photoconductance. **(B)** Sample activity from the local circuit model of panel **(A)**. Due to strong and delayed recurrent mutual inhibition, the network undergoes a collective oscillatory activity with a frequency in the gamma range. Even when oscillations at the population level are very regular (see an example “LFP”—i.e., average membrane potential—time series), individual neurons spike very irregularly with a much lower firing rate (see raster plot of the activity of 100 excitatory neurons).

To study the response to light stimulation of systems involving transfected neuronal areas, we simulated the activity of simple canonic circuits composed of just one local area or of two local areas mutually coupled with equal strength. Each area was modeled as a large network of randomly interconnected excitatory and inhibitory neurons. As shown in Figure 2A, a fraction of these excitatory and inhibitory model neurons were equipped with ChR2 photoconductances, inducing depolarization in response to simulated light stimulation.

For most of the analyses reported in this study, we adopted within each local area strong and delayed inhibition and a sufficiently strong background drive (see Table 2). With such a choice of parameters, local circuits underwent—through an “ING”-type (i.e., “interneuron-generated”) mechanism (Whittington et al., 2000; Brunel and Wang, 2003; Brunel and Hansel, 2006; Tiesinga and Sejnowski, 2009) a marked and persistent oscillatory activity, well visible in the traces of a LFP-like signal. The collective frequency of oscillation was in the gamma range. Since driving was provided by background Poisson noise, the spiking activity of individual neurons was very irregular and characterized by a weaker firing rate (cf. Figure 2B). Weak pairwise correlations between spike trains coexisted thus with stronger pairwise correlations between membrane potential fluctuations (Yu and Ferster, 2010; Battaglia and Hansel, 2011). While inhibitory connections were confined within each local area, excitatory neurons could additionally establish long-range connections between distant local areas (Figure 5A). In this case, the gamma oscillations generated by each local circuit were set into one of many possible multistable phase-locked states (Figure 5B).

**Figure 3:**
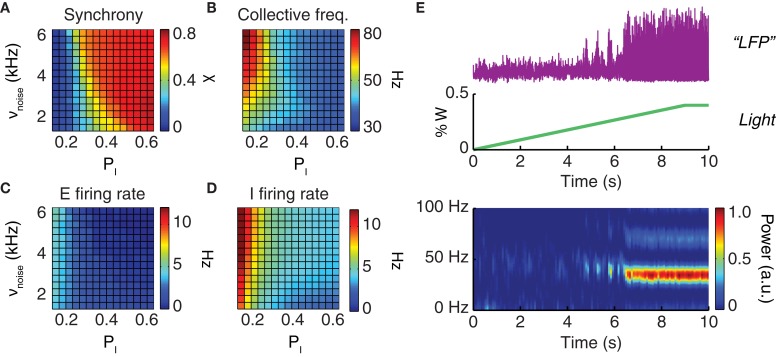
**Driving the network toward coherent oscillations.** The dynamical regime of a neuronal population depends on the strength of local inhibition (parameterized by the probability *p*_*I*_ of inhibitory connections) and on the strength of an external driving force (parameterized by the rate ν_noise_ of background inputs). Shown are the synchronization index **(A)** which has values in the unit interval (0 corresponds to asynchronous and 1 to perfectly synchronous dynamics); the oscillation frequency of collective activity **(B)**; and the average firing rates of excitatory **(C)** and inhibitory **(D)** neurons. All four quantities are presented in their dependence on the probability of inhibitory connections, *P*_*I*_, and the rate of background noise input, ν_noise_. **(E)** Constant or slowly ramping optogenetic stimulation increases the external drive to a neuronal population. This results in intensified collective oscillations and enhanced synchronization at the population level. From top to bottom: LFP time-series (purple) observed during a slowly ramping photostimulation (green); the associated spectrogram (graph at the bottom) indicates the development of highly coherent gamma oscillations as an effect of continuous photostimulation.

The dynamical features of the simulated neural activity, including in particular its degree of oscillatory synchrony, depended sensibly on the noisy drive to the network and on the strength of local inhibition. For increased drive intensity and/or stronger inhibitory interactions, a smooth transition occurred toward a dynamic regime characterized by elevated collective synchronization (Figure 3A). In this synchronous regime, the frequencies of the network oscillation were in the gamma range, varying between 40 and 70 Hz (Figure 3B), while the average firing rate of individual excitatory neurons varied between 1 and 3 Hz (Figure 3C) and of inhibitory neurons between 2 and 7 Hz (Figure 3D).

Starting from a very wide range of parameters including the probability of inhibitory connections and the strength of the external driving force (Figure 3), oscillatory synchrony can be robustly boosted by enhancing the external drive to the network. Qualitatively reproducing existing experimental findings (Adesnik and Scanziani, 2010; Akam et al., 2012), our simulations showed that slowly ramping or constant low-intensity optogenetic stimulation can be used to “switch on” a markedly oscillatory behavior. As shown by Figure 3E a network with poorly synchronous activity can be optogenetically driven toward higher oscillatory synchrony, as evident not only from LFP spectrograms but also visually from LFP traces.

In the following, we will mainly consider model networks tuned to generate strong LFP gamma oscillations. However, such a choice is not an arbitrary restriction. Indeed, high synchrony regimes—either spontaneously emergent or induced artificially by continuous photostimulation—are particularly suited for analyses of phase shifting and locking.

### Shifting the phase of an ongoing local oscillation

**Figure 4:**
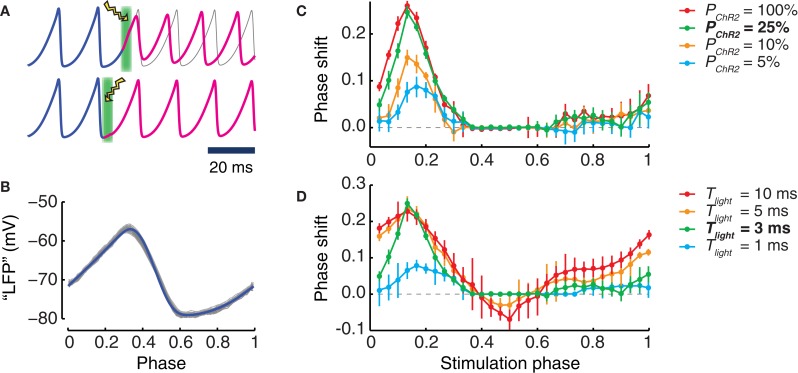
**Phase shifts induced by photostimulation. (A)** Examples of phase shifts induced by a single light pulse. Top: the phase (blue curve) of the oscillation of the transduced population is shifted by light perturbation (illustrated as a lightning symbol with green background) and, afterwards (magenta curve), remains advanced with respect to the unperturbed oscillation (gray curve). Bottom: such a phase shift cannot be seen when the timing of the light perturbation corresponds to other differently chosen oscillation phases. **(B)** Waveform of the oscillating LFP in dependence on the Hilbert phase. Shown are 500 oscillation cycles (gray) of a LFP and their average waveform (blue). By our conventions, the phase ranges in the unit interval. The maximum of the LFP is obtained for (Hilbert) phase values close to 0.3 while the minimum occurs for phase values close to 0.6. **(C,D)** phase shifts caused by light pulses applied at different (Hilbert) phases of the ongoing LFP oscillation. An optimal light intensity of 18% *W*_max_ is used. **(C)** Dependence of the phase shift on the transduction rate *P*_*ChR2*_ of the population (for a stimulus duration *T*_light_ = 3 ms). **(D)** Dependence of the phase shift on the stimulus duration *T*_light_ (for a fixed transduction rate of *P*_*ChR2*_ = 25%). Bold characters in the legend denote the “reference” phase shift, i.e., *P*_*ChR2*_ = 25% and *T*_light_ = 3 ms of stimulus duration (green curves). In panels **(C)** and **(D)**, error bars are standard deviation of the phase shifts obtained for different perturbations applied in a same time-bin.

It is well known that the effect of a perturbation to an oscillating system depends on the phase at which the perturbation is applied (Pikovsky et al., 2001). To explore the phase dependency of light stimulation, we applied simulated stimulation pulses with different durations *T*_light_ to local populations with different transduction rates *P*_*ChR2*_ (Figure 4). Light intensity was always set to the optimum value of *W*_light_ = 18% *W*_max_, which led to maximum evoked peak photocurrents.

For all the explored conditions, we always found strongest effects on the phase of an ongoing oscillation when the perturbation was applied at a phase half-way between the trough and the peak of the collective population oscillation (Figure 4B). In this case the phase of the perturbed oscillation was advanced with respect to the unperturbed case (Figures 4C,D). Short pulses lasting 1 or 3 ms led only to phase advance effects. As shown in Figure 4C, phase advances of the order of one quarter of a cycle could be achieved using such short pulses, over a very wide range of transduction rates, going from very high (100%) down to moderate (25%). Noticeable phase advance effects (although reduced to just one tenth of a cycle) could even be detected for transduction rates as low as 5%.

As displayed by Figure 4D, longer stimulation durations also led to phase lagging effects. These effects occurred in different ranges of perturbation application phases than for phase advancing effects. However, phase lagging effects were always weaker than phase advancing effects. For instance, for a transduction rate of 25%, pulses lasting 10 ms could induce phase advances of over a quarter of cycle, but only phase laggings of less than one tenth of cycle.

The positive peaks of the *phase response curves* (PRCs) plotted in Figures 4C,D were aligned across all conditions. The strongest phase shifting effects were always observed when the perturbation was applied in proximity of the phase ϕ = 0.17. We also mention that for the short stimulation duration used, the evoked photocurrent was dominated by the fast activation time-course. Inactivation played no role in determining the response. As a matter of fact, the effect of the fast initial rise of the photocurrent was to evoke a spike in the transduced neurons, as in panel 1A, and additional synchronous spikes evoked in a subpopulation of cells were the dynamic cause of the induced phase shift, as in Battaglia et al. (2012).

### Perturbing phase relations between different oscillating populations

**Figure 5:**
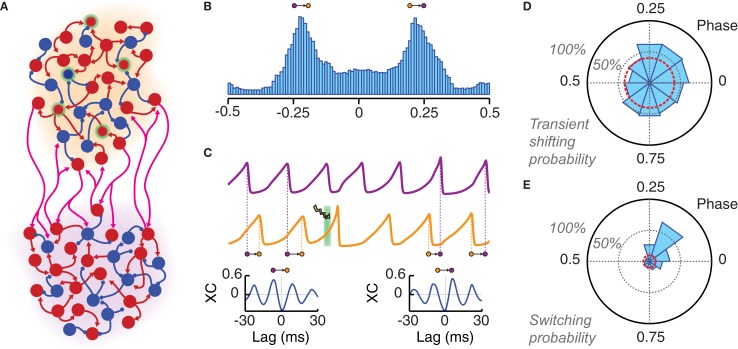
**Local photostimulation can reorganize long-range phase-locking patterns. (A)** Cartoon of two local populations (each of them with an individual background color: orange and violet) coupled by long-range excitatory connections. **(B)** Both populations oscillate in a non-regular way but with the same main frequency. A histogram of the instantaneous phase difference is shown for a pair of very long LFP time series (over 50,000 oscillation cycles). This distribution is clearly bimodal, indicating the existence of two favorite modes of approximate out-of-phase locking (with the orange population leading in phase over the violet, or the other way around). **(C)** LFP traces of two phase-locked populations. The application of a light pulse stimulation (denoted by a green background and a lightning symbol) can induce switching to another phase-locked mode. This is shown by the qualitative changes between the crosscorrelogram (XC, computed over 500 ms) of the two LFPs before (left) and after (right) light stimulation. Note the changed sign of the lag of the highest XC peak, which corresponds to an inversion of the direction of functional connectivity. **(D)** Probability of changing the average inter-population phase difference of more than 10% during five oscillation cycles after light stimulation (*P*_*ChR2*_ = 25%, *T*_light_ = 3 ms). This probability is presented by a polar histogram in dependence on the phase of the onset of the light stimulation (with respect to the leader population). The red circle indicates the probability of similarly large spontaneous phase shifts (i.e., without photostimulation). **(E)** Phase difference averaged over 50 cycles starting 10 cycles after the light pulse. A switching is considered as successful if the sign of this average phase difference has changed (see panel **B**). The probability of successful phase switching is given by a polar histogram, as in panel **(D)**. The red circle indicates the probability of spontaneous switching in the case of non-stimulated activity. Ignoring transient effects, switching can be induced with high probability only if the perturbation is applied within a specific narrow phase range.

After the controlled shifting of the phase of a local oscillation, we explored whether precisely phased stimulation could be used to manipulate phase relations between different local oscillating circuits. To do so, we considered a canonic circuit of two coupled oscillating areas, interconnected by long-range random excitatory projections (Figure 5A). In general, when driven into a synchronous regime, motifs of a few local areas mutually connected with equal strength can give rise to different phase-locked states. These states are associated to different patterns of inter-areal phase relations, which are maintained in a relatively stable manner over long time intervals (Battaglia et al., 2007, 2012).

The specific bi-areal network of Figure 5A generated two multi-stable phase-locked states. In the unperturbed system, background noise caused spontaneous switching between these two states (i.e., from one configuration of inter-areal phase relations to another). The result of these stochastic fluctuations was a clearly bimodal distribution of the instantaneous phase difference between the two areas (Figure 5B). The actual phase relations in the phase-locked modes depend ultimately on the PRC of the coupled populations. As discussed in Battaglia et al. (2007, 2012), the PRCs associated to our network model are such that they lead to *out-of-phase* locking for sufficiently strong inhibition (unless long-range synaptic delays are tuned *ad-hoc* within narrow intervals associated to in- or anti-phase configurations). Out-of-phase locking is found also in more general systems of pulse-coupled neurons (or neuronal masses) under certain conditions on synaptic delays (Woodman and Canavier, 2011; Wang et al., 2012).

In out-of-phase locked modes, it is always possible to identify one area (leader) whose oscillations lead in phase over the oscillations of the other area (laggard). This leads to anisotropic directed functional influences between local circuits (Battaglia et al., 2012), in agreement with the communication-through-coherence hypothesis (Fries, 2005), despite the fact that inter-areal connections are reciprocal and of equal strength in both directions. Switching between alternative phase-locking configurations would thus correspond to changes in the dominant direction of inter-areal functional influences. Spontaneous switching was a relatively rare event in the high synchrony regime explored here (the average waiting time for spontaneous switching was over 60 periods). Nevertheless, optogenetic stimulation could be used to actively trigger switching events (Figure 5C).

Inter-areal phase relations after the application of a single perturbation pulse were compared to the average locked phase difference before the pulse itself. We studied how both transient short-term and persistent long-term effects depend on the phase of perturbation onset. Figure 5D shows the probability that the average inter-areal phase difference for the five cycles directly following the perturbation has increased or reduced by at least 10% relative to the average phase difference prior to the perturbation. For a wide range of phases of stimulation onset, such probability was larger than 50% and remarkably larger than the level accounted for by spontaneous fluctuations of the inter-areal phase difference.

The dependency on the perturbation phase was more pronounced for long-term effects. Figure 5E shows the probability of a switch in phase locking, i.e., that the average inter-areal phase difference over a long time window beginning ten cycles after the perturbation has changed its sign (note, indeed, that the two phase-locked configurations of the simulated bi-areal motif are characterized by average phase-differences of Δϕ = ±Δϕ_locked_, cf. Figure 5B). In contrast to short-term shifting, the probability of actual switching was concentrated in a narrow phase interval centered on the peak of the single-area PRC, as expected from theory (Battaglia et al., 2012). The switching probability for other phase bins dropped quickly to the level of spontaneous switching.

Our simulations show that the peak probability of optogenetically-induced switching could rise above 60% even for small transduction rates of 25%. However, this happened only if the phase of the perturbation onset was precisely selected. Indeed, the comparison of Figures 5D,E shows that many of the short-term shifting effects observed for randomly phased perturbations did not develop into lasting changes in phase-locking. To conclude, we would like to mention that a similar pulse-induced reorganization of inter-areal phase relations could be achieved even when the perturbation was applied to the laggard rather than to the leader area [not shown, but see (Battaglia et al., 2012)].

### Closing the loop

**Figure 6:**
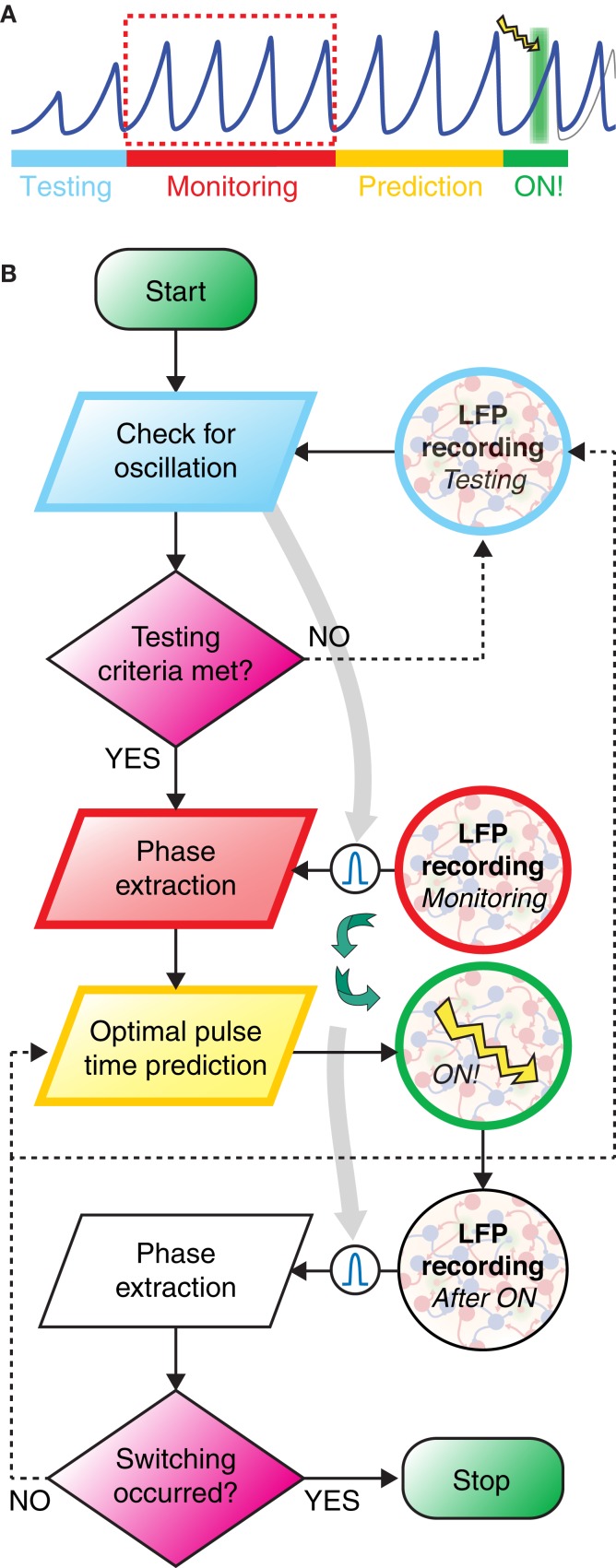
**Closed loop strategy for precisely phased photostimulation. (A)** Schematic illustration of the proposed experimental protocol. During the *testing stage* (light blue) the LFP is recorded and tested for sufficiently strong power in the gamma-range. If the gamma band power is high enough, then a bandpass-filter is tailored to its peak frequency (light gray arrow). In the *monitoring* stage (red), phases are extracted from the band-passed LFP. Based on these observations, during the *prediction stage* (yellow), lasting only a very few oscillation periods, a linear model of phase evolution is extrapolated to predict the time at which the target phase of the oscillation will occur next. A light pulse is then delivered at this predicted time (green background with lightning symbol). **(B)** The workflow of the closed loop experiment is presented as a flow chart, with the left swim lane presenting computation and decision steps and the right swim lane showing recording and stimulation of the transfected neuronal population. Curved green arrows highlight the closed-loop nature of the workflow, i.e., the light pulse stimulation delivered at a time depending on the phase evolution of LFP oscillations during the monitoring window.

As discussed in the last section, the controlled switching of inter-areal phase-locking—and, hence, of functional connectivity—required perturbations optimally phased with respect to ongoing oscillations. To increase the probability to induce switching, the timing of perturbation must thus be determined based on phase information extracted from recordings of the recent population activity. We suggest here a possible closed-loop protocol for the online prediction of the timing of stimulation achieving an optimal switching rate. The workflow of the proposed idealized experiment is outlined by a schematic time bar (Figure 6A) and a corresponding flow chart (Figure 6B). The potential performance of such protocol was studied by simulating the induction of switching in the bi-areal network of Figure 5A.

In contrast to this well behaved *in silico* model, oscillatory coherence *in vivo* or *in vitro* recordings is usually transient and confined to specific epochs. There is nevertheless experimental evidence that epochs of phase synchronization at fast gamma frequencies can persist over several hundreds of ms *in vivo* (Varela et al., 2001; Pesaran et al., 2002; Gregoriou et al., 2009; Bosman et al., 2012; Grothe et al., 2012). Detecting the onset of one of such oscillatory epochs was precisely the aim of the *testing stage*, in which LFPs in both areas of the bi-areal motif were recorded and their spectral characteristics extracted in real-time to verify that LFP power and inter-areal coherence with respect to a common frequency (band) rose above a minimum threshold (see section Materials and Methods).

The *monitoring stage* was entered immediately after the detection of an epoch of reliable inter-areal coherence. During this monitoring stage, LFP signals were recorded, filtered in real time through a low-order band-pass filter with a pass frequency optimized during the testing window and, finally, stored.

**Figure 7:**
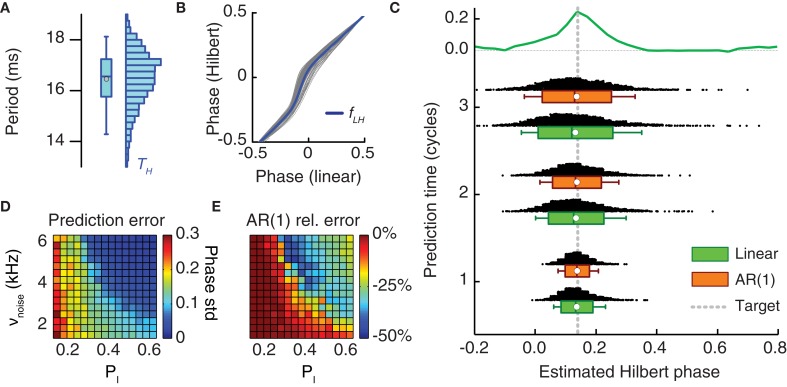
**Online prediction of the phase of stimulation onset. (A)** The period length of LFP oscillations fluctuates from cycle to cycle and has a broad-range uni-modal distribution (here shown for period lengths as estimated from the Hilbert phases). **(B)** Hilbert phase versus linear phase for a sample LFP time series. To speed-up the computation of *t*_ON_ in the prediction stage, the Hilbert phase can be approximated by a linear phase, since, as here shown, they are strongly correlated and the mild static non-linearity *f*_LH_ linking them can be neglected. **(C)** Distribution of the phase of *t*_ON_ predicted by two different methods and for different lengths of the prediction window (measured in oscillation cycles). Shown are histograms and box plots (box giving median and interquartile range, white circle the mean value and whiskers the 5-th and 95-th percentiles) of the predicted phase of light stimulation ϕ(*t*_ON_) for two prediction methods—pure linear extrapolation based on the average period length (green) and first order autoregressive [AR(1)] models (orange)—applied to period lengths recorded during the monitoring stage. Both the median and the mean of predicted Hilbert phase are in good agreement with the exact target phase (leading with highest probability to a phase shift) with a dispersion not larger than the width of the positive part of the reference phase-shift response curve (reproduced from Figures 4C,D on the top of the panel). **(D)** The prediction error (i.e., the standard deviation of the inferred phase ϕ(*t*_ON_) of photostimulation onset) depends on the synchronization level of the neuronal population activity (cf. Figure 3A). The prediction error based on linear extrapolation (measured in units of average oscillation period lengths) is shown for different probabilities of local inhibitory connection *p*_*I*_ and background noise rates ν_noise_. Larger synchronization leads to better prediction. **(E)** The ratio of the prediction error based on the AR(1) model and the prediction error based on linear extrapolation in dependence on the same parameters. For intermediate synchrony levels, the prediction error can be consistently reduced by the use of an AR(1) model.

A fast online analysis of the phase dynamics of the stored LFP of only the target area was then performed during the following *prediction stage*. Its aim was to predict the timing of one of the next occurrences of the target phase, solely from the phase information acquired during the monitoring stage. To keep the prediction window as short as possible, we propose to use computationally cheap and consequently linear techniques for phase extrapolation. Indeed, the “real” phase values (given by Hilbert Transform of the LFP signal, see section Materials and Methods) and a simple linear descriptor of the phase are strongly correlated (Figure 7B) and non-linear effects can be neglected in a first-order approximation.

The phase-locking between LFPs recorded after the stimulation application was finally compared with the locking existing before the stimulation to verify the successful induction of state switching.

Figure 7 analyzes the simulated performance of the proposed protocol, when applied to *in silico* recordings from the bi-areal network motif of Figure 5. Figure 7C shows how the predicted onset phases of light stimulation concentrate around the actual target phase given by the peak PRC value of ϕ_target_ = 0.18. The scattering of predicted phases is computed by hypothesizing prediction stages with different possible (short) durations. This estimate was done with two prediction schemes which both have fast implementations: a simple linear extrapolation based on the average period length and a first-order autoregressive model [AR(1)] (see section Materials and Methods), accounting for correlations between the durations of successive oscillation cycles, at least approximately. For increasing lengths of the prediction window, the median and the average value of the predicted Hilbert phase remained very close to the target (Figure 7C). However, the distribution of extrapolated phase values broadened, as indicated by their increasing dispersion. Nevertheless, for a prediction window lasting three oscillation cycles—a sufficiently long time to perform the fast computation required for linear extrapolation (see section Discussion)—the interquartile range of predicted phase values was still contained in the width of the reference PRC. Consequently, we still expect an enhanced effectiveness of light stimulation pulses applied at the inferred time *t*_ON_, compared to randomly timed pulses.

The error made in predicting a target phase depends necessarily on the quality of the recorded oscillation. The dynamical regime of the simulations in Figures 5 and 7C was strongly synchronous. As previously discussed, the degree of synchrony of the collective response depends on the external driving force to the network and on the strength of local inhibition (Figure 3A). We performed phase prediction based on recordings of simulated dynamics with different degrees of synchrony. As shown in Figure 7D, stronger synchrony was associated to smaller prediction errors. Interestingly, prediction errors remained moderate even when considering regimes “at the edge of synchrony.” Furthermore adopting a more elaborate AR(1) approach yielded the strongest performance improvement with respect to simpler linear extrapolation precisely for these intermediate synchrony values (Figure 7E).

In contrast, prediction errors associated to weak synchronous dynamics were larger and even the AR(1) approach failed to improve over linear extrapolation in these cases. However, in these regimes, the dynamics rarely displayed long-lasting oscillatory epochs and the probability of spontaneous switching was comparable to the one of induced switching, thus invalidating our analysis protocol. In these cases, therefore, continuous photo-stimulation should be used to enhance the degree of coherence of the coupled populations activity (analogously to Figure 3E).

## Discussion

### From power boosting to reliable phase control

Optogenetic stimulation has been successfully applied to boost the power of fast neural oscillations *in vivo* and *in vitro*. In Cardin et al. (2006), pulsed optogenetic stimulation *in vivo* was used to highlight the existence of a resonance at gamma range frequencies of local inhibitory cortical microcircuits. Adesnik and Scanziani (Adesnik and Scanziani, 2010) and Akam et al. (2012) experimented with ramped light stimulation to induce long-lasting oscillatory episodes in slices.

Beyond controlling oscillation power, the experiments by Akam et al. (2012) are closely related to the first part of our model study. They used 5 ms-long light stimulation pulses to shift local oscillation phases and quantify the phase response curves (PRCs) of oscillations in hippocampal slices, analogously to the simulated experiment of Figure 4. The hippocampal PRC measured by Akam et al. (2012) was distinctly biphasic, leading to phase advancement or phase delaying, depending on the phase of application of the stimulation. Such biphasic PRC shape is in qualitative and approximately in quantitative agreement with the PRCs extracted from our local population model for stimulation pulses of comparable lengths (cf. Figure 4D, orange curve for 5 ms-long pulses and red curve for 10 ms-long pulses).

Interestingly, however, the PRCs extracted from our model for shorter stimulation durations lacked phase-delaying regions and displayed only a narrow phase range leading to consistent phase advancement. Furthermore, they were characterized by a relatively broad range of application phases for which light stimulation was completely ineffective. These features of the PRC shapes are robustly obtained if the circuit mechanism for the generation of oscillations dominantly relies on delayed mutual interactions within interneuronal networks (Battaglia et al., 2007, 2012). One can actually use very different neuronal models to obtain oscillatory and phase-locking behaviors that qualitatively match those observed. For instance, spatially structured networks of integrate-and-fire neurons (Battaglia and Hansel, 2011) have dynamical regimes that tightly correspond to those of homogeneous networks of the conductance-based neurons (Battaglia et al., 2007) that we adopt here. We predict therefore that similarly looking PRCs could be obtained in the case of Kainate-induced *in vitro* oscillations in slices, in which excitatory neurons are entrained by a coherently oscillating interneuronal population but are not actively involved in the generation of the local rhythm (Fisahn et al., 2004; Bartos et al., 2007; Andersson et al., 2012).

Narrow phase ranges associated to large PRC values reduce the probability of inducing stable phase shifting by applying stimulation at arbitrary times. However such narrow intervals become a desirable resource when optogenetic stimulation is precisely phased conditional to ongoing oscillations, as executable in perspective with a closed-loop setup. Indeed, PRC shapes like the reference PRC discussed in Figure 3 (green curve for *P*_*ChR2*_ = 25%, and *T*_light_ = 3 ms light-pulses) could allow an “all-or-none” control of phase shifting, in which strong effects are obtained only if the stimulation is applied within a specific target range of phases, but in which undesired switching triggered by noise or by a misapplied input is largely suppressed.

### A simple ChR2 model captures non monotonic photoresponse

The light-activated cation channel ChR2 activates more rapidly and supports larger peak current amplitudes for increasing light intensities. Therefore, we speculated that brief, high intensity light pulses would provide the optimal stimulation for our purposes. To our knowledge there were no studies that systematically documented ChR2 current responses for stimuli with light intensities above 20 mW/mm^2^ (Ishizuka et al., 2006; Ernst et al., 2008; Lin et al., 2009). At this intensity the activation rate is still light sensitive and we aimed to increase it even more using light intensities as high as approximately 130 mW/mm^2^. While the activation rate did indeed decrease further, the fact that the peak current amplitude *decreased* for intensities above approximately 20 mW/mm^2^ came to us as a surprise (Figures 1B,C). This behavior has not been reported before, to the best of our knowledge, though the measurements published in Lin et al. (2009) hint at a decreasing peak amplitude for the highest intensity applied there, which was approximately 19.8 mW/mm^2^.

Such phenomenon might be reminiscent of the photoreactive P480b intermediate state, which can be converted by blue light to the early P500 intermediate state. This transition was proposed as a shortcut of the photocycle from a spectroscopic study of ChR2 channels (Ritter et al., 2008). Since previously published models of ChR2 currents (Nikolic et al., 2006, 2009) could not account for this non-monotonic light response, it was necessary to deploy a novel model. Our simple conductance-based model correctly captures the existence of an optimal light intensity for photostimulation, without need to incorporate elaborate details about the ChR2 molecular structure and dynamics. Note that the application of our model is not limited to brief light pulses, but can also predict light-induced conductance in response to ramps of light (cf. Figure 3E).

Our model is also accurately data-constrained. To calibrate model parameters, light induced changes of ChR2 conductance were measured in voltage clamp. If the voltage can be clamped throughout a cell, any changes in the whole-cell current can be attributed to ChR2 conductances. In differentiated neurons, however, this perfect voltage control cannot be attained. This is obvious from the recording in Figure 1A (black trace), where the activation of ChR2 depolarized the axon sufficiently to activate voltage-dependent sodium channels, which created an unclamped spike. Even when sodium channels are blocked, the conditions are not optimal for a precise biophysical characterization. Using essentially passive and electrotonically compact cells, such as HEK-293 cells (Nikolic et al., 2009), provided optimal recording conditions (Figure 1B). The smaller amplitude of the photocurrents in these cells reflected differences in cell surface and expression levels, while the biophysical properties of ChR2 were most likely identical to those expressed in neurons.

### Technical feasibility

As discussed above, the extraction of PRCs describing the collective response of a transduced neuronal population to light stimulation was already achieved *in vitro* (Akam et al., 2012). Our modeling study suggests that a similar approach could be successfully applied *in vivo*, since phase-shifting effects can be robustly obtained with high and low transduction rates, covering the wide range achievable with different experimental techniques (Adamantidis et al., 2007; Petreanu et al., 2007; Wang et al., 2007; Takahashi et al., 2012). The success rate will depend on a suitably tuned light intensity and on the ability to select the phase of the stimulation onset conditional on ongoing oscillation dynamics. Another factor that might enhance the controllability of phases is the use of faster variants of Chr2, such as ChETA (Gunaydin et al., 2010) and the E123T/T159C (Berndt et al., 2011) mutants.

A closed-loop approach is required for determining the optimal timing of pulse stimulations. Figure 7C shows that if the time required for the prediction stage is of the order of a few oscillation cycles, then the discrepancy between the target and the actual perturbation phase is comparable to the width of the peak of the PRC. Consequently the resulting phase shifting should remain close to the optimum. The prediction strategy that we propose (Figure 6) is based uniquely on a small number of linear computations, which are particularly suited for ultrafast (millisecond scale) implementation on reconfigurable hardware chips (Zhuo and Prasanna, 2008; Sadrozinski and Wu, 2011) or on GPU architectures (Owens et al., 2008; Volkov and Demmel, 2008) on which FFT algorithms can be efficiently implemented (Bhattacharyya et al., 2010). As a matter of fact, hardware implementations of period extraction (Waskito et al., 2010) and autoregressive modeling of biologic signals (Marinkovic et al., 2005; Kim and Rosen, 2010) have already proven to be order(s) of magnitude faster than on conventional CPUs. Taking into account these high levels of performance and the approximations we propose to implement, a length of the prediction window of ~50 ms that corresponds to approximately three cycles of a 40–70 Hz rhythm appears completely realistic.

Our simulated oscillations constitute an idealized model for neuronal rhythms measured *in vivo* or *in vitro*. In our model, especially when the synchronization index is very high, cycle-to-cycle period length fluctuations are positively correlated with weak to intermediate correlation strength. In real neuronal oscillations, however, adaptation or other phenomena might introduce more complex correlation patterns between the lengths of different periods. Nevertheless, such correlations might still be captured by AR(1) modeling, as hinted to by the better performance of AR(1) in dynamic regimes at the “edge of synchrony” (Figure 7E), in which period length fluctuations are more strongly correlated.

Under specific experimental conditions, long-lasting oscillatory epochs might be a rare event. It would then become difficult to meet the conditions for the applicability of our protocol (i.e., the testing stage of Figure 6 might never be passed). In this case, continuous optogenetic stimulation could be used to stabilize and boost oscillations, as simulated in Figure 3E. Then, similarly to the approach of Akam et al. (2012), precisely timed “kicks,” superposed on this continuous light stimulation, could be used to perturb the instantaneous phase. In this sense, optogenetic stimulation is more promising than electric micro-stimulation. First, it allows combining continuous and pulsed stimulation within a single setup. Second, it can control with high selectivity the degree of synchronization, not only by providing an unspecific drive to the entire network, but also enhancing the drive to specific neuronal subpopulations, like for instance FS-PV cells which provide the phasic inhibition crucial for rhythm generation (Cardin et al., 2006; Sohal et al., 2009).

Finally, we are optimistic that the network models of transduced neural populations that were pioneered by Talathi et al. (2011) and further developed in this study are powerful tools, which will be increasingly adopted to conduct, optimize and accelerate the design and the calibration of closed-loop optogenetic experimental protocols.

### Probing phase-coding and communication-through-coherence

Reliable optogenetic manipulation of the phase dynamics of oscillating neuronal populations would open the way to an interventional exploration of phase coding schemes. In the phase coding framework, it is argued that the phase of spikes relative to a “reference clock”—paced either by a stimulus-locked (De Charms and Merzenich, 1996; Arabzadeh et al., 2006) or an internally-generated oscillation (O'Keefe and Recce, 1993; Siegel et al., 2009)—carry information, which is independent from and multiplexed with the one conveyed by rate fluctuations (Montemurro et al., 2008). Anticipating or delaying the ticks of such a “reference clock,” as the one putatively framed by slow cortical oscillations (Kayser et al., 2012), should perturb the decoding of phase-based representations.

Beyond the control of the phase of a local oscillation, inter-areal phase correlations could be disrupted transiently by unspecific optogenetic stimulation (Figure 5D). Furthermore, precisely-phased perturbations determined within a closed-loop system could induce persistent switching between alternative phase-locked dynamic patterns (Tiesinga and Sejnowski, 2010; Battaglia et al., 2012). In this sense, the realization of an experiment inspired by the idealized analysis of Figure 4, would provide a direct testing of the communication-through-coherence hypothesis (Fries, 2005). More specifically, it would allow experimental testing of whether different sets of inter-areal phase relations lead to different inter-areal functional interactions and to an altered balance between bottom-up and top-down information flows, as predicted by theory (Battaglia et al., 2012).

A reorganization of phase relations between distant neuronal populations might have perceptual or behavioral consequences. Selective alteration of inter-population phase relations, for instance between areas FEF and V4 (Gregoriou et al., 2009) or areas V1 and V4 (Grothe et al., 2012), might be used to suppress or boost attentional effects or even to emulate reorienting of attention. Furthermore, our theoretical investigations suggest that stimulation applied locally to a single area might induce distributed reorganization of phase relations between other more distant areas (Battaglia et al., 2012). Closed-loop optogenetic stimulation might then in perspective be used to trigger system-level switching between global brain states (Deco et al., 2009; Freyer et al., 2011).

## Author contributions

Agostina Palmigiano and Demian Battaglia performed the simulations. Annette Witt, Agostina Palmigiano, and Demian Battaglia analyzed the simulations. Andreas Neef and Ahmed El Hady performed and analyzed the experiments. Annette Witt, Andreas Neef, Fred Wolf, and Demian Battaglia designed the models and the study. Annette Witt, Agostina Palmigiano, Andreas Neef, Ahmed El Hady, Fred Wolf, and Demian Battaglia wrote the manuscript.

### Conflict of interest statement

Ahmed El Hady is also appointed editor of the present special research topic issue. The other authors declare that the research was conducted in the absence of any commercial or financial relationships that could be construed as a potential conflict of interest.
